# O-Serotype Conversion in *Salmonella* Typhimurium Induces Protective Immune Responses against Invasive Non-Typhoidal *Salmonella* Infections

**DOI:** 10.3389/fimmu.2017.01647

**Published:** 2017-12-04

**Authors:** Pei Li, Qing Liu, Hongyan Luo, Kang Liang, Jie Yi, Ying Luo, Yunlong Hu, Yue Han, Qingke Kong

**Affiliations:** ^1^Institute of Preventive Veterinary Medicine, College of Veterinary Medicine, Sichuan Agricultural University, Chengdu, China; ^2^Center for Infectious Diseases and Vaccinology, The Biodesign Institute, Arizona State University, Tempe, AZ, United States; ^3^College of Animal Science and Technology, Southwest University, Chongqing, China; ^4^Department of Infectious Diseases and Pathology, University of Florida, Gainesville, FL, United States

**Keywords:** *S*. Typhimurium, *S*. Enteritidis, *S*. Choleraesuis, *S*. Newport, O-antigen, live attenuated *Salmonella* vaccine, cross-protection

## Abstract

*Salmonella* infections remain a big problem worldwide, causing enteric fever by *Salmonella* Typhi (or Paratyphi) or self-limiting gastroenteritis by non-typhoidal *Salmonella* (NTS) in healthy individuals. NTS may become invasive and cause septicemia in elderly or immuno-compromised individuals, leading to high mortality and morbidity. No vaccines are currently available for preventing NTS infection in human. As these invasive NTS are restricted to several O-antigen serogroups including B1, D1, C1, and C2, O-antigen polysaccharide is believed to be a good target for vaccine development. In this study, a strategy of O-serotype conversion was investigated to develop live attenuated *S*. Typhimurium vaccines against the major serovars of NTS infections. The immunodominant O4 serotype of *S*. Typhimurium was converted into O9, O7, and O8 serotypes through unmarked chromosomal deletion–insertion mutations. O-serotype conversion was confirmed by LPS silver staining and western blotting. All O-serotype conversion mutations were successfully introduced into the live attenuated *S*. Typhimurium vaccine S738 (Δ*crp* Δ*cya*) to evaluate their immunogenicity in mice model. The vaccine candidates induced high amounts of heterologous O-polysaccharide-specific functional IgG responses. Vaccinated mice survived a challenge of 100 times the 50% lethality dose (LD_50_) of wild-type *S*. Typhimurium. Protective efficacy against heterologous virulent *Salmonella* challenges was highly O-serotype related. Furthermore, broad-spectrum protection against *S*. Typhimurium, *S*. Enteritidis, and *S*. Choleraesuis was observed by co-vaccination of O9 and O7 O-serotype-converted vaccine candidates. This study highlights the strategy of expressing heterologous O-polysaccharides *via* genetic engineering in developing live attenuated *S*. Typhimurium vaccines against NTS infections.

## Introduction

Salmonellae are facultative intracellular pathogens that are capable of infecting a wide range of animals and are responsible for high mortality and morbidity worldwide (1, 2). More than 2,500 *Salmonella* serovars have so far been identified, while 99% of human and animal infections are caused solely by one subspecies, *Salmonella enterica* subsp. *enterica* (*S. enterica*) (3). With regard to human disease, *S. enterica* has traditionally been divided into a small number of human-restricted invasive typhoidal *Salmonella* and thousands of non-typhoidal *Salmonella* (NTS) (4). Human host-restricted *S. enterica* serovars Typhi and Paratyphi A are the leading causes of typhoid and paratyphoid enteric fevers, respectively, while NTS predominantly cause a self-limiting gastroenteritis in healthy individuals. Although NTS generally produce diarrhea, they can become invasive and cause septicemia, as well as focal infections such as meningitis, endocarditis, and osteomyelitis (5). Typically, these invasive NTS are restricted to several O-antigen serogroups, including B1, D1, C1, and C2. In Sub-Saharan Africa, invasive NTS (iNTS) have emerged as a prominent cause of bloodstream infections (6), with *S. enterica* serovars Typhimurium (serogroup B1) and Enteritidis (serogroups D1) being the most prevalent. Clinical diagnosis in these regions is difficult, as there are no signs or symptoms to distinguish NTS from a number of other common infections, such as endemic malaria (7). In developed countries, NTS infection is mainly foodborne and causes gastroenteritis, with bacteremia typically occurring as a rare complication associated with immunodeficiency. Serogroup C is becoming the most common serogroup in the USA and has been increasing in Europe over the last decade (8). Additionally, increasing frequencies of multi-drug resistance among invasive isolates threaten the effectiveness of amenable antibiotic treatments (9). To date, vaccines are regarded as the most economical and effective ways to prevent salmonellosis (10, 11).

The first clinical vaccine against *Salmonella* was an inactivated whole-cell vaccine (TAB vaccine), which was used extensively by the British and US military to prevent typhoid fever and associated deaths (11, 12). However, these vaccines are no longer used due to their high reactogenicity (13). As an improvement, the following three types of vaccines have been licensed: the live attenuated vaccine Ty21a, a purified unconjugated Vi polysaccharide, and a Vi polysaccharide conjugated to tetanus toxoid (14). Except for *S*. Typhi, no vaccines against other *Salmonella* serovars are currently licensed for use in humans. Vaccines against NTS are even further behind in the development pipeline. Studies of NTS vaccines are mainly focused on live attenuated *Salmonella* vaccine and O-antigen polysaccharide-based subunit vaccine.

Live attenuated vaccines have a number of potential advantages, including an excellent ability to elicit T-cell responses, a convenient oral vaccination route, and good capacities to induce mucosal immunity (15, 16). The major challenge in developing live attenuated vaccines lies in attaining an optimal level of attenuation without compromising immunogenicity (17). To date, the only live attenuated NTS vaccine that has completed a Phase 1 study is WT05, a *S*. Typhimurium vaccine containing attenuated *aroC* and *ssaV* (18), though other live NTS vaccine candidates are in preclinical development. These include *S*. Typhimurium and *S*. Enteritidis, lacking the *guaBA* and *clpP* genes, that were shown to protect mice against a lethal homologous challenge (19). Attenuation strategies target global regulators of gene expression, such as mutations in *cya* and *crp*, are also promising (20, 21). Another potential strategy is to introduce mutations in *Salmonella* that lead to regulated delayed attenuation *in vivo via* dependence on key nutrients that are not available in host tissues, thus leading to attenuation after invading and colonizing host (22, 23). However, vaccines based on these strategies have not yet reached clinical trials.

O-antigen polysaccharide, a portion of lipopolysaccharide, is responsible for *Salmonella* serovar specificity and is considered to be an excellent protective antigen (24, 25). Many efforts have been made to develop vaccines that contain repeating O-polysaccharide polymers conjugated to a range of protein carriers (10) including tetanus toxin (TT), diphtheria toxin (DT), and the non-toxic recombinant form of DT (CRM197) (10, 11). The conjugation of *Salmonella* O-polysaccharide to *Salmonella* proteins is likely to be a more effective alternative to exogenous carriers (26, 27), as they could induce immune responses against two *Salmonella* antigens instead of one. However, these attenuated NTS vaccines and glycoconjugate vaccines are largely restricted to a single serovar or group of serovars, mainly against serovars Typhimurium and Enteritidis and unable to or only partially provide cross-protection against heterologous *Salmonella* infections. Moreover, these serovar-specific vaccines may suffer the potential risk of changing the epidemiology of NTS. The clinical evidence has shown that *S*. Choleraesuis (group C) is becoming prevalent in the USA and Europe (8). However, little work has been done to develop vaccines against *Salmonella* serogroup C infections (28, 29).

O-antigens of *Salmonella* groups B and D share a common trisaccharide backbone of α-Man(1→4)-α-Rha-(1→3)-α-Gal-(1→2), which serologically contributes to epitopes 1 and 12 (30). In each case, a unique dideoxyhexose sugar contributes to their immunodominance in serogroup specificity, namely, O4 (group B, α-Abe(1→3)Man), O8 (group C2, α-Abe(1→3)Rha), and O9 (group D, α-Tyv(1→3)Man). The unique sugar components and linkages in the O-unit of group C1 contribute to factor O7 (31). The O-antigenic characteristics of *S*. Typhimurium, *S*. Enteritidis, *S*. Choleraesuis, and *S*. Newport are hereafter referred to as O4, O9, O7, and O8, rather than their full O-antigen formulae. Passive protection studies demonstrated that IgG or IgM directed against the immunodominant group-specific epitope O4 played more important for protection than antibodies specific to epitope O1 and O12 (32). Immunodominant O-antigens also play an important role in eliciting protective memory responses against *Salmonella* (33). In this study, we modified *S*. Typhimurium O-antigen structure, converting its native B1 group immunodominant O4 serotype to the D1 group O9, C1 group O7, and C2 group O8 *via* chromosomal genetic manipulation. We expect to combine these heterologous O-polysaccharides in live attenuated *S*. Typhimurium vaccines to protect against serogroups B, D, C1, and C2, thereby preventing the majority of NTS infection. Our work highlights the possibilities of achieving a broad protective coverage against *S*. Typhimurium, *S*. Enteritidis, *S*. Choleraesuis and *S*. Newport by live attenuated *S*. Typhimurium vaccines based on O-serotype conversion.

## Materials and Methods

### Bacteria, Plasmids, Media, and Growth Conditions

All bacteria and plasmids used in this study are listed in Table 1. The O-serotype-converted *Salmonella* mutants were all derived from wild-type *S*. Typhimurium S100. *Escherichia coli* and *S. enterica* were grown at 37°C in Luria-Bertani (LB) broth or in LB agar. *sacB* gene-based counter selection in allelic exchange experiments was performed on LB agar containing 10% sucrose with no sodium chloride added and grown at 30°C (34). Media were supplemented with 25 µg/ml chloramphenicol for selection. Diaminopimelic acid (50 µg/ml) was added for the growth of the Δ*asd* strain (35). Electrocompetent *E. coli* or *S. enterica* cells were prepared as described previously (36). *In vitro* growth rates of *Salmonella* strains were determined by optical density measurements.

**Table 1:** Bacterial strains and plasmids used in this study.

Strains or plasmids	Description^a^	Source
***Salmonella* and *Escherichia coli***		
S100	*S*. Typhimurium, O4	(47)
S246	*S*. Enteritidis, O9	(47)
S340	*S*. Choleraesuis, O7	(47)
S264	*S*. Newport, clinical isolate from cattle, O8	IPVM
S1031	Δ*abe-1:prt-tyv*_D1_, O9	This study
S1124	Δ(*rmlB*-*wbaP*)*3*:(*wzy*_C1_-*wzx*_C1_), O7	This study
S1131	Δ(*wzx*_B1_-*wabN*)*2*:(*wzx*_C2_-*wbaZ*), O8	This study
S738	Δ*crp*-*24* Δ*cya*-*25*, O4	(21)
S1075	Δ*abe*-*1*:*prt*-*tyv*_D1_ Δ*crp*-*24* Δ*cya*-*25*, O9	This study
S1157	Δ(*rmlB*-*wbaP*)*3*:(*wzy*_C1_-*wzx*_C1_) Δ*crp*-*24* Δ*cya*-*25*, O7	This study
S1116	Δ(*wzx*_B1_-*wabN*)*2*:(*wzx*_C2_-*wbaZ*) Δ*crp*-*24* Δ*cya*-*25*, O8	This study
χ7232	*E. coli endA1 hsdR17* (r_K_−, m_K_+) *glnV44 thi-1 recA1 gyrA relA1 Δ*(*lacZYA-argF*)*U169* λ*pir deoR* (ϕ*80dlac* Δ(*lacZ*)*M15*)	(40)
χ7213	*E. coli thi-1 thr-1 leuB6 glnV44 fhuA21 lacY1 recA1 RP4-2-Tc*:Mu λ*pir* Δ*asdA4* Δ*zhf-2*:Tn*10*	(40)
**Suicide plasmids**		
pYA4278	*sacB* mobRP4 R6K *ori* Cm+	(38)
pSS241	Δ*pagL7* construction	(43)
pSS908	Δ*abe*-*1* construction	This study
pSS022	Δ*crp*-*24* construction	(21)
pSS023	Δ*cya*-*25* construction	(21)
pSS916	Δ*abe*-*1*:*prt*-*tyv*_D1_ construction	This study
pSS937	Δ(*rmlB*-*wbaP*)*3* construction	This study
pSS971	Δ(*wzx*_B1_-*wbaN*)*2* construction	This study
pSS1009	Δ(*rmlB*-*wbaP*)*3*:(*wzy*_C1_-*wzx*_C1_)	This study
pSS1010	Δ(*wzx*_B1_-*wabN*)*2*:(*wzx*_C2_-*wbaZ*)	This study

*IPVM, Institute of Preventive Veterinary Medicine at the Sichuan Agricultural University in China*.

*^a^The O-antigen serotype information for each applicable strain only showed its immunodominant O-serotype*.

### Recombinant DNA Techniques

DNA manipulations were performed using standard methods (37) and were approved by Division of Environmental Health and Safety of Sichuan Agricultural University. No restriction endonuclease sites were introduced when amplifying DNA fragments from chromosomes or plasmids. DNA concentrations and purity were measured using a Nanodrop ND-2000 spectrophotometer (Thermo Fisher Scientific). DNA fragments were assembled using Gibson Assembly Master Mix according to the manufacturer’s instructions (New England Bio Labs). PCR was performed in 20 µl reaction volumes in a Thermo Scientific Arktik Thermal Cycler. Reaction mixtures contained one volume of 1 µM (each) forward and reverse primers and DNA template (~50 ng plasmid DNA or ~100 ng chromosomal DNA) with an equal volume of 2× premix PrimeSTAR Max DNA polymerase (TaKaRa). Thermal cycler conditions were 98°C for 2 min; 30 cycles of 98°C for 1 min, 55°C for 30 s, and 72°C for 15–30 s/kb; and a final extension at 72°C for 10 min.

### Suicide Vector Construction and Genetic Manipulation in *S*. Typhimurium

**Figure 1:**
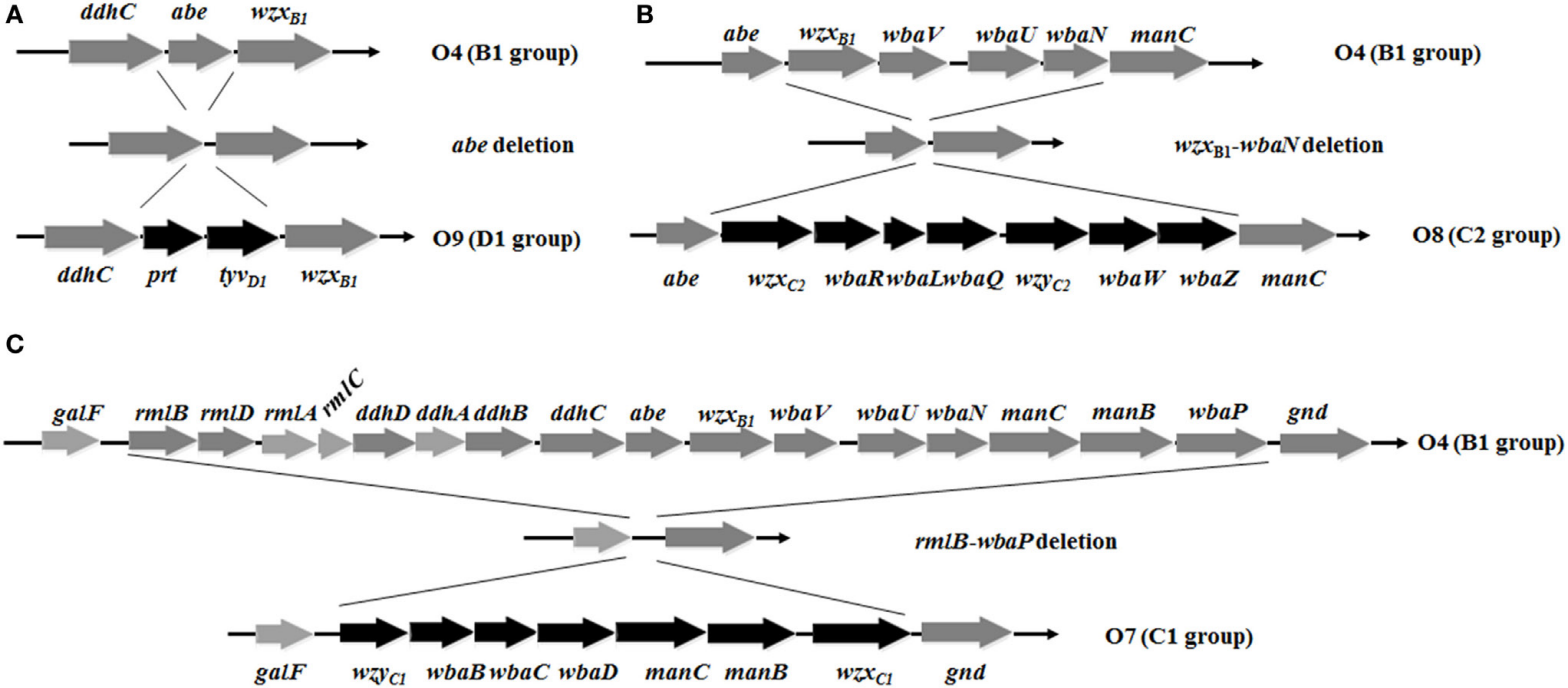
Deletion-insertion mutations resulting in O-serotype conversion in *Salmonella* Typhimurium. **(A)** The *abe* gene was deleted and inserted by *prt*-*tyv*_D1_ from *S*. Enteritidis, converting the O4 serotype to O9. **(B)** Genes *wzx*_B1_, *wbaV, wbaU*, and *wbaN* were deleted and inserted by *wzx*_C2_, *wbaR, wbaL, wbaQ, wzy*_C2_, *wbaW*, and *wbaZ* from *S*. Newport, converting the O4 serotype to O8. **(C)** The entire O-antigen gene cluster of *S*. Typhimurium was deleted and inserted by the C1 serogroup O-antigen gene cluster from *S*. Choleraesuis, converting the O4 serotype to O7. Genes inserted into the *S*. Typhimurium O-antigen gene cluster for O-serotype conversion are indicated by black arrows and native genes are depicted as gray arrows. Diagrams were drawn to scale.

All primers used in this study are listed in Table S1 in Supplementary Material. We used a *sacB* gene-based suicide vector to counter-select deletion and/or insertion mutations. For deletion mutation suicide vector construction, two homologous DNA fragments, upstream and downstream of the gene or operon to be deleted, were amplified using primer pairs designated D-N-1F/D-N-1R and D-N-2F/D-N-2R (N represents the name of the gene or operon to be deleted). After DNA purification, these two fragments were fused by PCR using primer pairs designated D-N-1F/D-N-2R and linked into the suicide vector pYA4278 (38). For insertion mutation suicide vector construction, the allelic gene or operon to be inserted in a directed site was amplified using primer pairs designated (G)In-I-F/(G)In-I-R (I represents the name of the gene or operon to be inserted). The backbone of the suicide vector, which contained the homologous upstream and downstream sequence of the site to be inserted, was amplified using primer pairs designated (G)Vec-D-N-F/(G)Vec-D-N-R. All purified DNA fragments were assembled in order using Gibson Assembly (39), resulting in targeted gene or operon inserted into the directed site in a suicide plasmid. All allelic gene exchanges or whole-operon replacement in *S*. Typhimurium were achieved in two steps (Figure 1): deletion of the original gene or operon followed by insertion of the target gene or operon. Chromosomal modifications were introduced by suicide plasmids using standard methods (40, 41). All of the constructed mutants, either intermediates or final constructs, were routinely sequenced.

### LPS Silver Staining and Western Blotting

LPS were prepared and visualized by the method of Hitchcock and Brown (42). LPS samples were separated *via* 12.5% SDS-PAGE gels and transferred to nitrocellulose membranes using a Trans-Blot SD semidry transfer system (Bio-Rad, Hercules, CA, USA). Membranes were first incubated with O-antigen signal-factor rabbit antisera (BD Biosciences) or vaccinated murine pooled sera (1:100 dilution) followed by secondary anti-rabbit or anti-mouse horseradish peroxidase-conjugated antibody (Sigma) at a 1:1,000 dilution. Patterns were detected by chemiluminescence using western ECL blotting substrates (Bio-Rad).

### P22 Transduction Studies

P22HT *int* was propagated on S. Typhimurium S100 carrying the chromosomal integrated suicide vector pSS241 (43), which confers chloramphenicol resistance. Strains to be tested were grown overnight in LB broth at 37°C. Cultures were diluted 1:100 into fresh LB broth and grown at 37°C to an OD_600_ of 0.6. Then, 10 µl of phage (1 × 10^8^ PFU) was mixed with 1 ml of bacteria (~5 × 10^6^ CFU) and incubated at 37°C for 30 min. After the incubation, the mixture was centrifuged and resuspended in 1 ml of PBS. A 100-µl aliquot was spread onto LB agar plate containing 25 µg/ml chloramphenicol. Colonies were counted after an overnight incubation at 37°C.

### Motility Assay

Motility assays were performed on LB plates containing 0.3% agar. The plates were allowed to dry at room temperature for approximately 2 h before the assays. Then, 6 µl of freshly grown bacteria (~5 × 10^6^ CFU) was spotted onto the middle of the plates and incubated at 37°C for 6 h. The diameters of the colonies (in millimeters) were measured.

### Minimum Inhibitory Concentration (MIC) Test

The MICs of deoxycholate (DOC) and polymyxin B were determined using 96-well microtiter plates. Two-fold serial dilutions of DOC (0.39–59 mg/ml) and polymyxin B (0.078–10 µg/ml) were made along the plates. Bacteria were grown to an OD_600_ of 0.6 and diluted to ~5.0 × 10^4^ CFU/ml in LB broth. Then, 100 µl of diluted bacteria suspension was added to each well and followed by an overnight incubation at 37°C. The optical density of each well was determined using an iMark™ Microplate Reader (Bio-Rad, Hercules, CA, USA). The threshold of inhibition was 0.1 at OD_600_.

### Attachment and Invasion Assay

The human epithelial type 2 (Hep-2) cell line (ATCC strain CCL-6) was used to perform bacterial attachment and invasion assays as described previously (44). The bacteria were added to each well at a multiplicity of infection of 10:1. The percentage of attachment rate was calculated as follows: percentage of attachment = 100 × (number of cell-associated bacterial/initial number of bacterial added). The percentage of invasion rate was calculated as follows: percentage of invasion = 100 × (number of bacteria resistant to gentamicin/initial number of bacteria added).

### Virulence Determination and Colonization in Mice

All animal studies were conducted in compliance with the Animal Welfare Act and regulations stated in the Guide for the Care and Use of Laboratory Animals, which was approved by Sichuan Agricultural University Institutional Animal Care and Use Committee (Ya’an, China; Approval No. 2011028).

Six-week-old female BALB/c mice were purchased from Dashuo Biotechnology Co., Ltd. (Chengdu, China). To determine the 50% lethality dose (LD_50_), bacteria were grown statically overnight at 37°C. Overnight cultures were diluted 1:100 into fresh LB media, grown at 37°C until reaching an OD_600_ of 0.8–0.9. Cells were harvested by centrifugation at 3,452 × *g* at room temperature, washed once, and adjusted to the required inoculum density in buffered saline with gelatin (BSG). Groups of six mice each were infected orally with 20 µl of BSG containing various doses of S. Typhimurium S100 or its derivatives, ranging from 1 × 10^4^ to 1 × 10^9^ CFU. Animals were observed for 4 weeks after infection, and deaths were recorded daily. The LD_50_ for each strain was calculated using the method of Reed and Muench (45). To evaluate colonization, groups of three mice were orally inoculated with 20 µl of BSG containing 1 × 10^9^ CFU bacteria. On days 4 and 8 post-inoculation, Peyer’s patches, spleen, and liver samples were collected. Samples were homogenized, and dilutions were plated onto MacConkey and LB agar to determine viable counts.

### Immunization and Measurement of Immune Response

Groups of 12 mice each were inoculated orally with 20 µl of BSG containing approximately 1 × 10^9^ CFU vaccine strains on day 0 and boosted on day 14 with the same dose. Blood samples were collected after 28 days. Mice were challenged orally on day 56 with 5 × 10^7^ CFU of *S*. Typhimurium, *S*. Choleraesuis, or *S*. Enteritidis (~100 times LD_50_).

*S*. Typhimurium and *S*. Enteritidis LPS were purchased from Sigma (St. Louis, MO, USA). *S*. Choleraesuis and *S*. Newport LPS were purified as described previously (46). A quantitative enzyme-linked immunosorbent assay (ELISA) was performed to determine serum antibody concentrations with the following modifications. Microtiter plates were coated with *Salmonella* LPS. The capture antibody, unlabeled goat antimouse IgG (H + L) (BD Pharmingen, San Diego, CA, USA) at 1 µg/ml in PBS, was added to extra uncoated wells to generate the standard curve. The plates were incubated overnight at 4°C, followed by blocking with PBS containing 5% BSA for 1 h at room temperature. For the LPS-coated wells, 100 µl of diluted serum was added to individual wells in triplicate. For the capture antibody-coated wells, the purified mouse IgG standard (for the standard curve quantification, BD Pharmingen, San Diego, CA, USA) was added, followed by two-fold serial dilutions starting at 0.5 µg/ml. The plates were incubated for 1 h at 37°C and then treated with biotinylated goat anti-mouse IgG (Southern Biotechnology Associates, Birmingham, AL, USA). The wells were developed with a streptavidin-alkaline phosphatase conjugate (Southern Biotechnology Associates, Birmingham, AL, USA), followed by a *p*-nitrophenylphosphate substrate (Sigma-Aldrich, St. Louis, MO, USA). Color development was recorded at 405 nm using an iMark™ Microplate Reader (Bio-Rad, Hercules, CA, USA). The ELISA standard curve was drawn using Curve Expert software (Hyams DG, Starkville, MS, USA). Serum antibody concentrations were calculated based on absorbance values and the standard curve.

### Complement Deposition Assay

Sera used for complement deposition assays were pooled sera taken from mice after the second immunization and were heated at 56°C for 30 min to inactivate endogenous complement. Bacteria were grown to an OD_600_ of 0.8 and harvested by centrifugation at 6,000 rpm for 2 min. Bacterial pellets were washed, centrifuged, and resuspended to approximately 5 × 10^8^ CFU/ml in PBS. Then, 20 µl of bacterial sample was incubated with 80 µl of complement-inactivated sera at 37°C for 30 min. Bacteria were then washed once with PBS, resuspended and incubated with 100 µl of fresh naive BALB/c mouse sera at 37°C for 30 min. After another wash with PBS, the samples were incubated with 100 µl of FITC-conjugated goat anti-mouse complement C3c (Abcam) at a dilution of 1:100 on ice for 30 min in the dark. After incubation, the bacteria were washed with PBS, resuspended in 1% formaldehyde, and latter analyzed with a flow cytometer (BD FACSVerse™). The negative control was wild-type *S*. Typhimurium incubated with non-vaccinated complement-inactivated mice sera, and the positive control was wild-type *S*. Typhimurium incubated with complement-inactivated rabbit anti-O4 *Salmonella* sera (BD Biosciences). All other processes were the same as the test groups.

### Analysis of Differential Uptake of *Salmonella* by Macrophages

An *in vitro* assay was performed to analyze the differential uptake of *S*. Typhimurium, *S*. Enteritidis, *S*. Choleraesuis, and *S*. Newport by the RAW264.7 macrophage cell line. Briefly, 1 × 10^5^ RAW264.7 cells in Dulbecco’s modified Eagle’s medium (DMEM) containing 10% FBS (Newborn calf serum) and Pen/Strep were allowed to adhere to a 24-well plate for 24 h. Each well contained approximately 5 × 10^5^ RAW264.7 cells. Approximately 30 min prior to infection, the old media were replaced with fresh DMEM containing only 10% FBS. In each well, 5 × 10^6^ *Salmonella* opsonized with relative vaccinated mice pooled sera or naive serum (1 µl serum for a 100-µl volume of *Salmonella* in PBS) were added. Gentamicin was added to each well at a final concentration of 100 µg/ml at different time intervals of 0, 20, 40, and 60 min, and the plates were incubated for 1 h to kill non-phagocytized bacterial cells. After three washes with PBS, the macrophages were lysed with 1% Triton X-100 and appropriate dilutions were plated on LB agar plates. Colonies were enumerated the next day.

### Statistical Analysis

Data were analyzed using the GraphPad Prism 5 software (Graph Software, San Diego, CA, USA) by one-way or two-way ANOVA followed by Tukey’s multiple-comparison post-test. Kaplan–Meier survival curve comparisons were calculated by comparing two groups at each time point through the log-rank (Mantel–Cox) test. The data were expressed as the mean ± SEM. *P* < 0.05 was considered statistically significant.

## Results

### O-Serotype Conversion in *S*. Typhimurium

**Figure 2:**
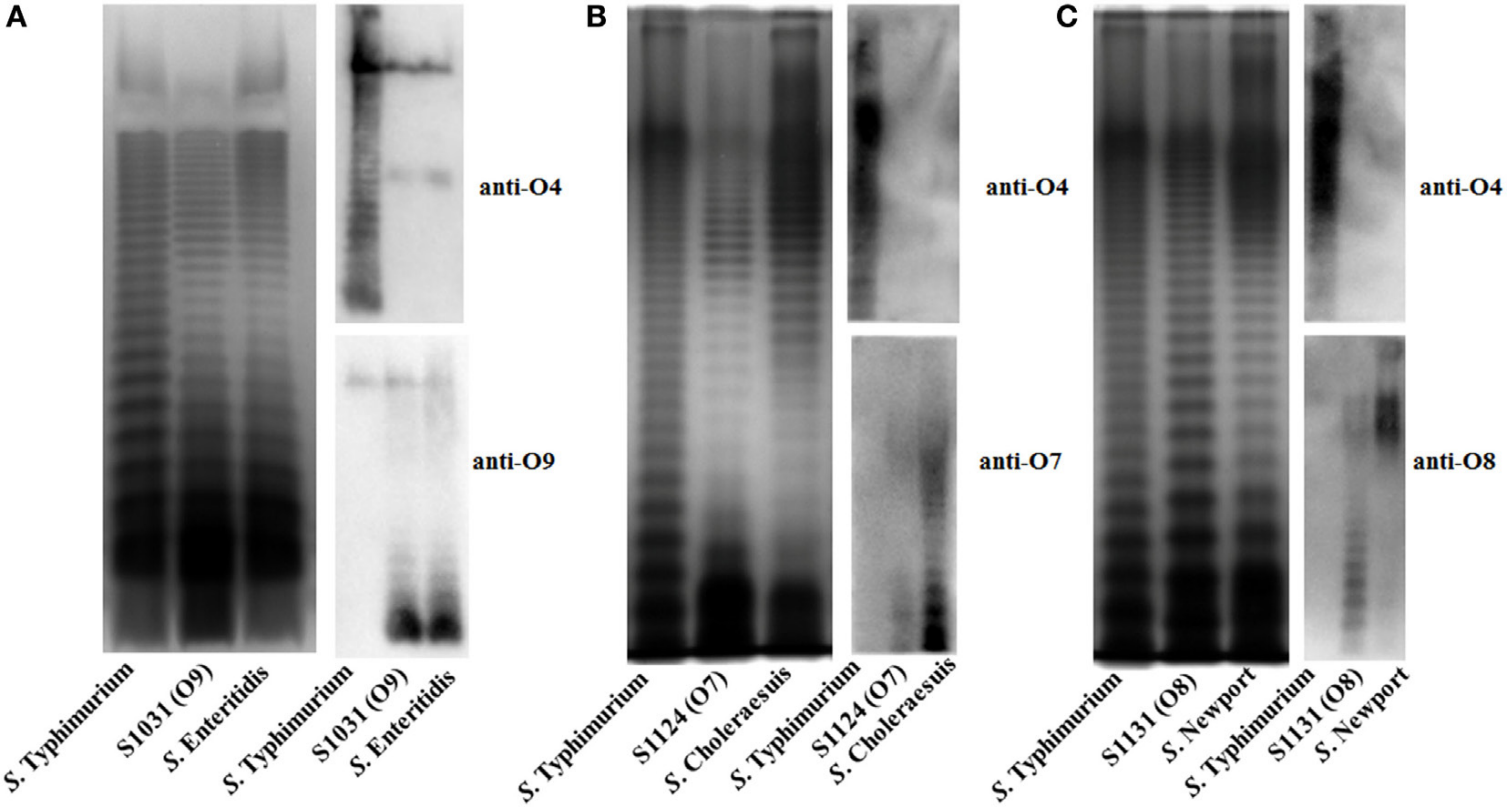
LPS profiles of O-serotype-converted mutants. **(A)** The LPS profiles of wild-type *Salmonella* Typhimurium, *S*. Enteritidis. and S1031 (O9) were compared. The O-serotype of S1031 was confirmed by incubation with *Salmonella* D1 serogroup-characterized O9 single-factor antisera, as indicated. **(B)** The LPS profiles of wild-type *S*. Typhimurium, *S*. Choleraesuis, and S1124 (O7) were compared. The O-serotype of S1124 was confirmed by incubation with *Salmonella* C1 serogroup-characterized O7 single-factor antisera, as indicated. **(C)** The LPS profiles of wild-type *S*. Typhimurium, *S*. Newport, and S1131 (O8) were compared. The O-serotype of S1131 was confirmed by incubation with *Salmonella* C2 serogroup-characterized O8 single-factor antisera, as indicated.

To develop effective live attenuated vaccines against invasive NTS infections, we targeted the structurally hyper-variable O-antigens. The distinctive O-antigen gene clusters of groups B1, D1, C1, and C2 were compared (Figure S1 in Supplementary Material), together with their chemical structures, to identify the relevant sugar components and glycosidic linkages (Figure S2 in Supplementary Material). The genetic modifications we used to achieve the desired *S*. Typhimurium O-serotype conversions are shown in Figure 1. Specifically, (1) the *abe* gene in *S*. Typhimurium was replaced with *prt-tyv*_D1_ from *S*. Enteritidis to convert the O4 serotype to O9, resulting in S1031 (Δ*abe*:*prt*-*tyv*_D1_) (O9) (Figure 1A); (2) the genes *wzx*_B1_-*wbaN* were replaced with *wzx*_C2_-*wbaZ* from *S*. Newport to convert O4 into O8, resulting in S1131 [Δ(*wzx*_B1_-*wabN*):(*wzx*_C2_-*wbaZ*)] (O8) (Figure 1B); and (3) the entire O-antigen gene cluster of group B1 was replaced with C1 from *S*. Choleraesuis to convert O4 into O7, resulting in S1124 [Δ(*rmlB*-*wbaP*):(*wzy*_C1_-*wzx*_C1_)] (O7) (Figure 1C). The LPS profiles of all these O-serotype-converted mutant strains were examined by silver staining and confirmed by western blotting (Figure 2). Notably, the LPS profiles of S1031 and S1131 differed from their parent *S*. Typhimurium, but exhibited similar patterns to *S*. Enteritidis (Figure 2A) and *S*. Newport (Figure 2C), respectively. However, the LPS profile of S1124 matched neither that of *S*. Typhimurium nor that of *S*. Choleraesuis (Figure 2B), but western blotting showed that S1124 generated LPS reactive with anti-O7 factor serum, indicating that O-antigen polysaccharide of *S*. Choleraesuis was successfully produced and ligated to *S*. Typhimurium core moiety in S1124 (Figure 2B). These O-serotype conversion mutations were later introduced into a live attenuated *S*. Typhimurium vaccine strain, S738 (Δ*crp* Δ*cya*) (O4), resulting in S1075 (Δ*abe*:*prt*-*tyv*_D1_ Δ*crp* Δ*cya*) (O9), S1157 [Δ(*rmlB*-*wbaP*):(*wzy*_C1_-*wzx*_C1_) Δ*crp* Δ*cya*] (O7), and S1116 [Δ(*wzx*_B1_-*wabN*):(*wzx*_C2_-*wbaZ*) Δ*crp* Δ*cya*] (O8), respectively, for further evaluation of immunogenicity and protective efficacy.

### *In Vitro* Characterization of O-Serotype-Converted Mutants

*In vitro* assays were done in triplicate. Phage P22 infections were performed to further examine the O-antigen structure of the mutants. The mutants were grown in LB broth and used as recipients for transduction assays. The number of transductions obtained from S1031 (O9) was similar to that from wild-type S100. However, we did not obtain any transductions from S1124 (O7) or S1131(O8) (Table 2).

**Table 2:** Transduction efficiencies, minimum inhibitory concentration (MIC) of deoxycholate (DOC) and Polymyxin B, swimming motility, and virulence of wild-type *Salmonella* and its derivatives.

Strain	Serotype changed^a^	Number of P22 transductants^b^	MIC		Swimming motility (mm)^c^	LD_50_ (CFU)
			DOC (mg/ml)	Polymyxin B (μg/ml)		
S1031 (O9)	O9	539 ± 37	25	0.625	34.01 ± 2.057	1.07 × 10^7^
S1124 (O7)	O7	0	25	0.625	38.53 ± 1.862	1.10 × 10^7^
S1131 (O8)	O8	0	25	0.625	41.97 ± 0.548	1.83 × 10^7^
*S*. Typhimurium S100	O4	586 ± 44	25	1.25	41.43 ± 0.129	1.59 × 10^5^
*S*. Enteritidis S246	O9	633 ± 35	25	1.25	40.17 ± 0.321	5.12 × 10^5^
*S*. Choleraesuis S340	O7	0	25	1.25	41.22 ± 0.457	4.95 × 10^4^
*S*. Newport S264	O8	0	25	1.25	40.82 ± 0.252	>10^9^

*DOC, deoxycholate*.

*^a^O-serotype conversion in S. Typhimurium*.

*^b^The phage lysate used for transduction was grown on a chloramphenicol-resistant strain. Transduction was performed as described in Section “Materials and Methods.” The results reflect the numbers of chloramphenicol-resistant colonies obtained after transduction (means ± SD)*.

*^c^The average diameter in millimeters (mean ± SD)*.

We next evaluated the impact of the O-antigen modifications on virulence and survival attributes. The mutants were evaluated for their sensitivity to the bile salt DOC and the cationic antimicrobial peptide polymyxin B. The DOC MICs did not differ among these strains, whereas the polymyxin B MICs for wild-type S100 were twofold higher than those for S1124 (O7), S1031 (O9), and S1131 (O8) (Table 2). We observed slightly slower growth rates for S1031 (O9), S1124 (O7), and S1131 (O8) compared to wild-type S100, but the differences were not significant (Figure S3 in Supplementary Material). All of the mutants retained wild-type or near wild-type motility (Table 2). To be effective, a live attenuated *Salmonella* vaccine needs interact with host epithelial cells. Thus, we examined the ability of our *Δcya Δcrp* derivatives to attach to and invade Hep-2 cells. No significant differences among strains were observed (Figure S4 in Supplementary Material).

### Virulence and Colonization of the Mutants in BALB/c Mice

Wild-type *S*. Typhimurium S100, *S*. Enteritidis S246, and *S*. Choleraesuis S340 displayed high virulence, with LD_50_ values of approximately 10^5^ CFU (47), whereas the LD_50_ of wild-type *S*. Newport S264 was greater than 10^9^ CFU, indicating a non-virulent phenotype of S264 in the murine model. The LD_50_ values of S1031 (O9), S1124 (O7), and S1131 (O8) were of a similar order of magnitude, approximately 10^7^ (Table 2). The colonization of each *Δcya Δcrp* vaccine candidate in murine Peyer’s patches, spleens, and livers was determined on days 4 and 8 after oral inoculation. All of the candidates displayed good colonization in Peyer’s patches, livers, and spleens, and no significant differences were observed among these groups. No deaths occurred during this period (Figure S5 in Supplementary Material).

### Immune Responses Induced by Live Attenuated Vaccines

**Figure 3:**
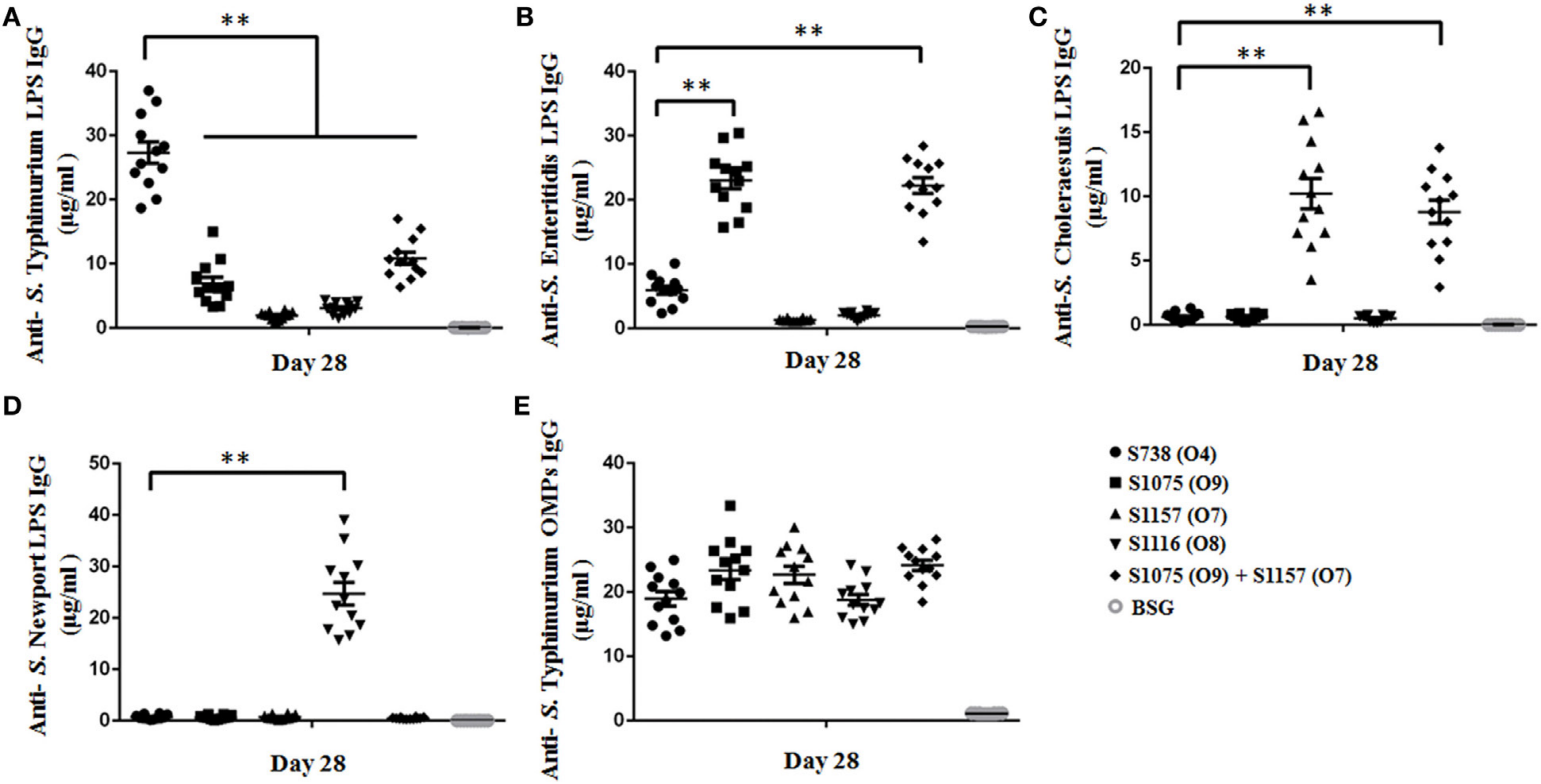
Sera IgG responses. Anti-*Salmonella* Typhimurium **(A)**, anti-*S*. Enteritidis **(B)**, anti-*S*. Choleraesuis **(C)**, and anti-*S*. Newport **(D)** LPS IgG antibody concentrations in vaccinated mice sera were measured. **(A)** Anti-*S*. Typhimurium LPS IgG levels induced by the parent strain S738 (O4) were significantly higher than those induced by other vaccine candidates (**, *P* < 0.01). **(B)** Anti-*S*. Enteritidis LPS IgG levels induced by S1075 (O9) were significantly higher than those induced by the parent strain S738 (O4) (**, *P* < 0.01). **(C)** Anti-*S*. Choleraesuis LPS IgG levels induced by S1157 (O7) were significantly higher than those induced by the parent strain S738 (O4) (**, *P* < 0.01). **(D)** Anti-*S*. Newport LPS IgG levels induced by S1116 (O8) were significantly higher than those induced by the parent strain S738 (O4) (**, *P* < 0.01). **(E)** There were no significant differences in anti-*S*. Typhimurium OMP IgG levels among the different groups of vaccinated mice sera. Antibody concentrations were calculated using a standard curve. All concentrations of the measured samples were within the range of the standard curve. Error bars represent the SEs of the means.

To assess the immunogenicity of these vaccine candidates, mice were inoculated orally with approximately 10^9^ CFU of each strain on day 0 and boosted on day 14 with the same doses. Anti-*S*. Typhimurium, anti-*S*. Enteritidis, anti-*S*. Choleraesuis, and anti-*S*. Newport LPS serum antibodies were measured on day 28. The results are depicted in Figure 3. Mice vaccinated with S1075 (O9) mounted a significantly higher anti-*S*. Enteritidis LPS immune response than those vaccinated with S738 (O4). A similar result was observed in mice vaccinated with S1157 (O7) or S1116 (O8), which mounted significantly higher anti-*S*. Choleraesuis or anti-*S*. Newport LPS immune responses, respectively, than those vaccinated with S738 (O4). All vaccines induced a significantly higher IgG2a response than IgG1. The low level of IgG1/IgG2a ratio indicated that the cellular immunity was biased to Th1-type immune response (Figure S6 in Supplementary Material), consistent with our and other previous observations that *Salmonella* induced a predominant Th1-type response to either heterologous antigens or *Salmonella* own antigens (48, 49). Negative control groups (BSG) did not mount a detectable immune response. Apart from ELISA, we also performed western blotting to evaluate the sensitivity of polyclonal antibodies using pooled sera from vaccinated mice (Figure S7 in Supplementary Material). The sera from S738 (O4)- and S1075 (O9)-vaccinated mice were cross-reactive to LPS from *S*. Typhimurium and *S*. Enteritidis, while those from S1157 (O7)- and S1116 (O8)-vaccinated mice were specific to LPS from *S*. Choleraesuis and *S*. Newport, respectively. No positive bands were detected using pooled sera from the BSG control group.

### C3 Complement Deposition and Opsonophagocytosis

**Figure 4:**
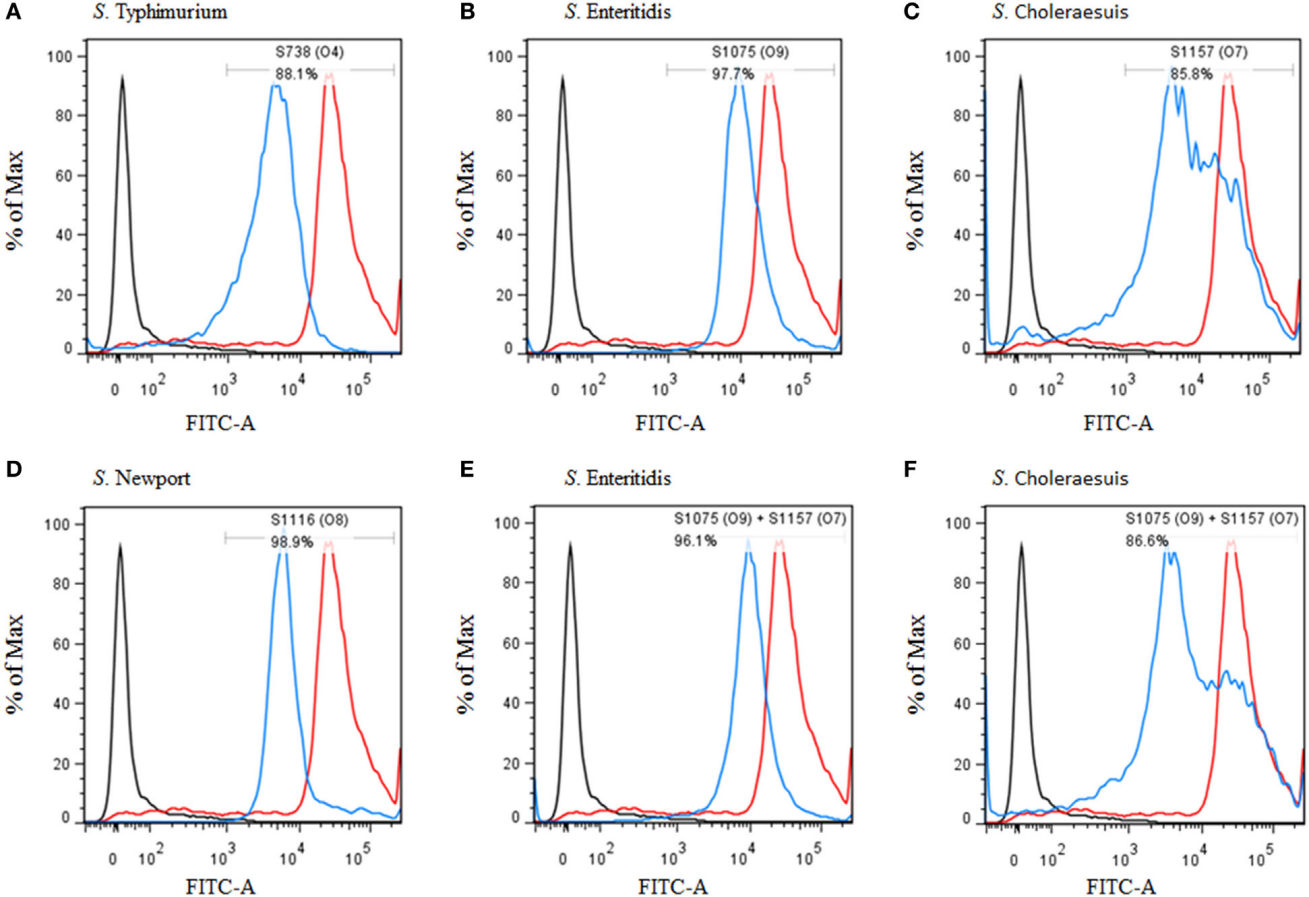
Flow cytometry histograms of C3 complement deposition. The percentage of FITC-positive bacteria was determined by flow cytometry. **(A)** Wild-type *S*. Typhimurium was incubated with sera from mice vaccinated with S738 (O4). Approximately 88.1% of bacteria were positive. **(B)** Wild-type *S*. Enteritidis was incubated with sera from mice vaccinated with S1075 (O9). Approximately 97.7% of bacteria were positive. **(C)** Wild-type *S*. Choleraesuis was incubated with sera from mice vaccinated with S1157 (O7). Approximately 85.8% of bacteria were positive. **(D)** Wild-type *S*. Newport was incubated with sera from mice vaccinated with S1116 (O8). Approximately 98.9% of bacteria were positive. **(E)** Wild-type *S*. Enteritidis was incubated with sera from mice co-vaccinated with S1075 (O9) and S1157 (O7). Approximately 96.1% of bacteria were positive. **(F)** Wild-type *S*. Choleraesuis was incubated with sera from mice co-vaccinated with S1075(O9) and S1157 (O7). Approximately 86.6% of bacteria were positive. The negative control (dark line) was wild-type *S*. Typhimurium incubated with sera from non-vaccinated mice, and the positive control (red line) was wild-type *S*. Typhimurium incubated with O4 single-factor rabbit antisera.

**Figure 5:**
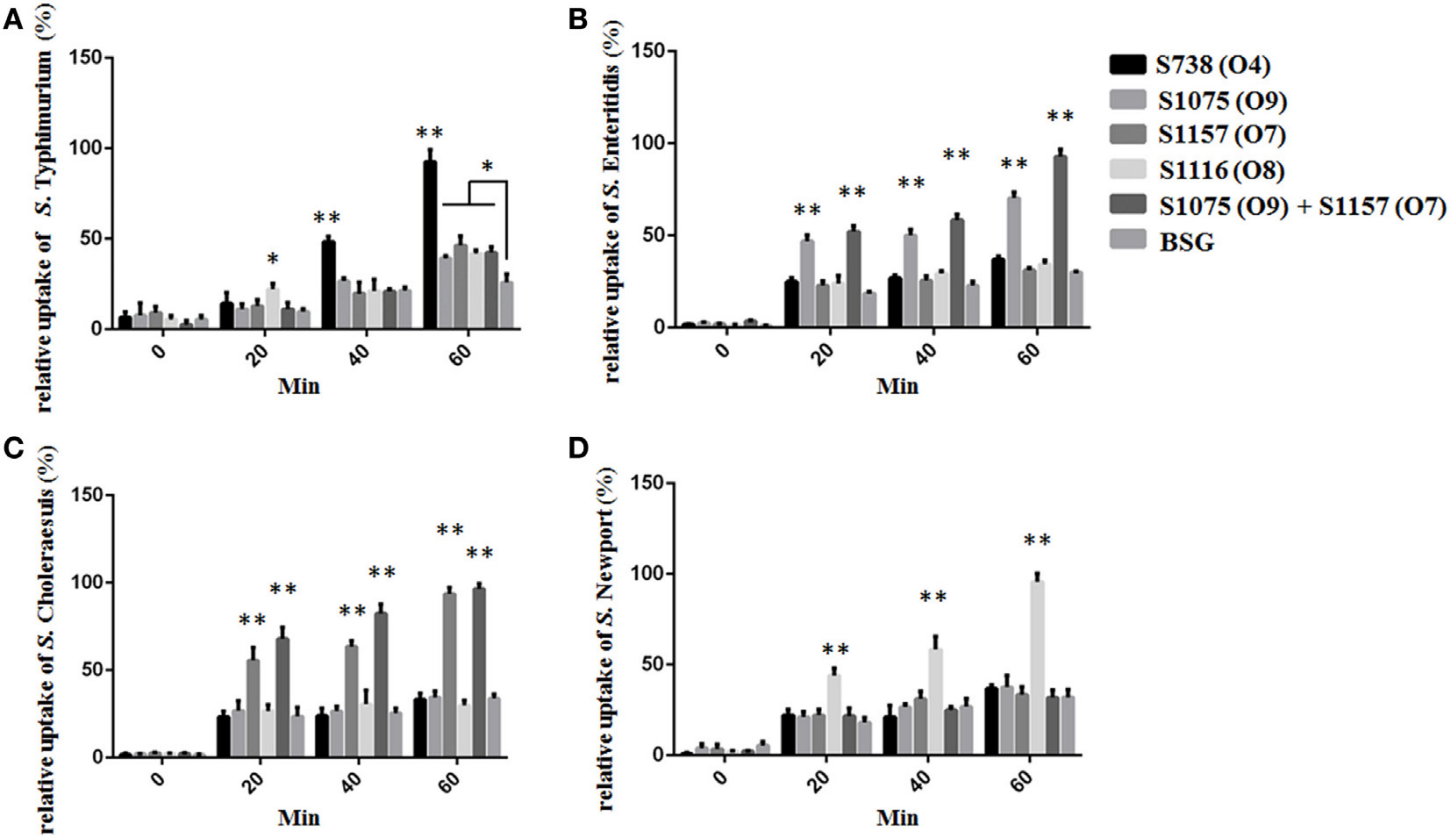
The differential uptake of *Salmonella* by RAW264.7 cells after serum opsonization. Compared to cells from control groups (*Salmonella* opsonized with naive serum), the uptake of *S*. Typhimurium **(A)**, *S*. Enteritidis **(B)**, *S*. Choleraesuis **(C)**, and *S*. Newport **(D)** opsonized with sera from mice primed with S738 (O4), S1075 (O9), S1157 (O7), and S1116 (O8), respectively, was significantly higher (**, *P* < 0.01). The number of enumerated cells was normalized to 100% for the maximal value. Error bars represent SEs of the means.

C3 complement deposition is the key process for antibodies targeting surface antigens, leading to complement activation and subsequent serum bactericidal activity. Therefore, we determined the ability of serum antibodies from vaccinated mice to direct complement deposition on the surface of different wild-type *Salmonella*. Sera used in this assay were boosted pooled sera from mice vaccinated with S738 (O4), S1075 (O9), S1157 (O7), S1116 (O8), and equal-volume-mixed S1075 (O9) and S1157 (O7). The percentage of bacteria coated with C3 was determined by flow cytometry (Figure 4). Compared to negative controls, a high percentage of bacteria deposited with C3 complement on the surfaces of wild-type *S*. Typhimurium (Figure 4A), *S*. Enteritidis (Figures 4B,E), *S*. Choleraesuis (Figures 4C,F), and *S*. Newport (Figure 4D) were detected when incubated with mice sera induced by S738 (O4), S1075 (O9), S1157 (O7), and S1116 (O8), respectively. These results indicated that antibodies in mice sera induced by live vaccine candidates were able to trigger the classical pathway of complement activation. Furthermore, an *in vitro* assay was performed to analyze the differential uptake of *S*. Typhimurium, *S*. Enteritidis, *S*. Choleraesuis, and *S*. Newport by RAW264.7 macrophages. The aim of this assay was to evaluate the role of vaccine-induced antibody opsonization in the early stages of opsonophagocytosis. Inoculation with sera primed with a specific O-serotype-converted vaccine resulted in significantly increased uptake of the same O-serotype wild-type *Salmonella* by macrophages (Figure 5). The uptake of *S*. Enteritidis inoculated with sera from mice primed with S1075 (O9) was significantly higher than naive sera or other non-specific sera. Similar results were observed with *S*. Choleraesuis and *S*. Newport when opsonized with sera from mice primed with S1157 (O7) and S1116 (O8), respectively. In particular, the uptake of both *S*. Enteritidis and *S*. Choleraesuis by macrophages was significantly increased when opsonized with sera from mice co-vaccinated by S1075 (O9) and S1157 (O7).

### Protective Efficacy of Live Attenuated Vaccines

**Figure 6:**
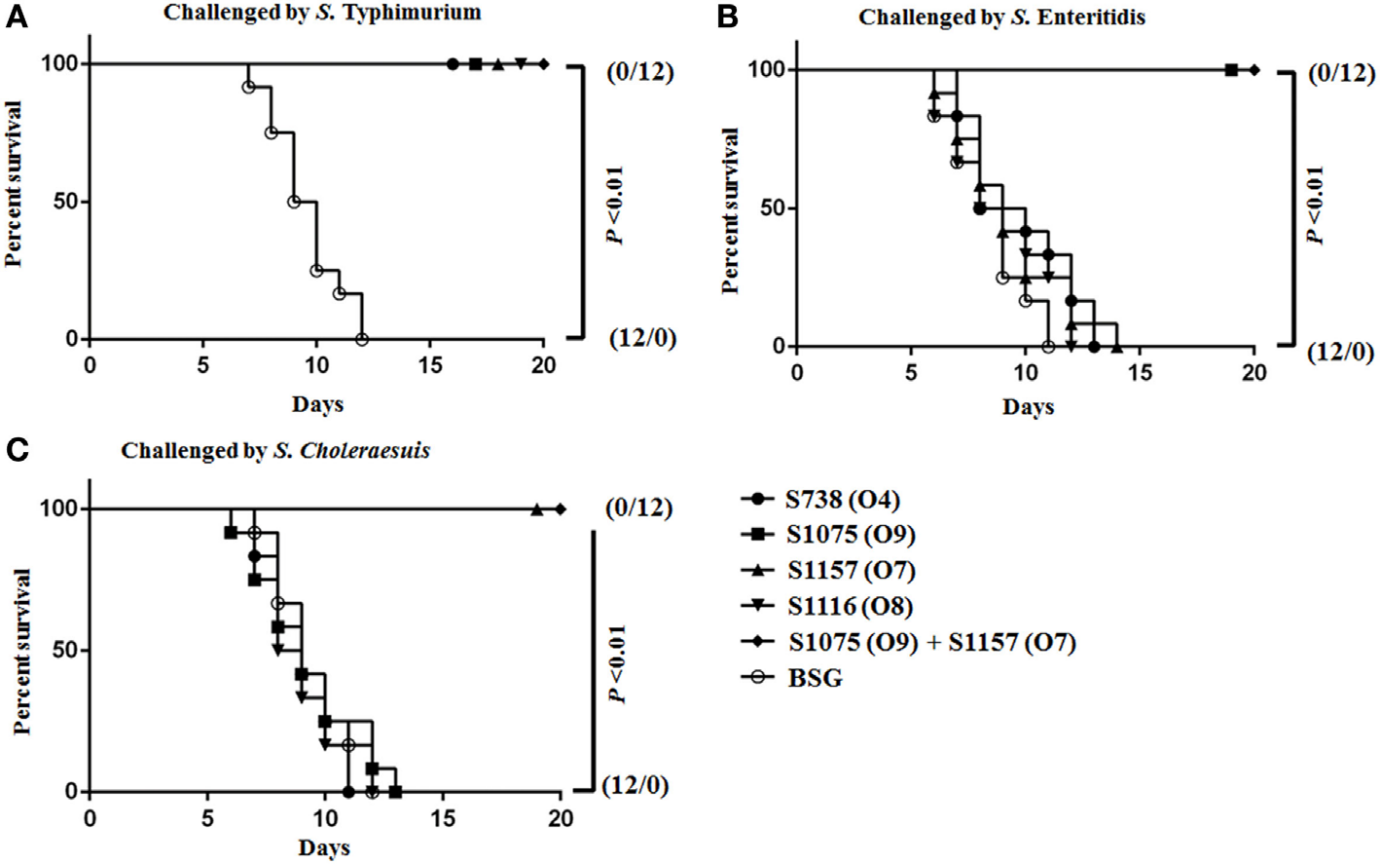
Survival curves after orally challenged by wild-type virulent *Salmonella*. Eight weeks after vaccination, BALB/c mice were challenged by about 100 times the LD_50_ of wild-type virulent *S*. Typhimurium **(A)**, *S*. Choleraesuis **(B)**, and *S*. Enteritidis **(C)**. *P* < 0.01, for all marked group versus BSG group.

Vaccinated mice were challenged orally on day 56 with a dose 100 times the LD_50_ of *S*. Typhimurium, *S*. Choleraesuis, and *S*. Enteritidis to evaluate protective efficacy. When challenged with *S*. Enteritidis and *S*. Choleraesuis, 100% protection was observed in mice vaccinated with S1075 (O9) (Figure 6B) and S1157 (O7) (Figure 6C), respectively. Complete protection was also observed in all vaccinated mice when challenged with *S*. Typhimurium (Figure 6A). As wild-type *S*. Newport was non-lethal in mice at an oral challenge dose of 10^9^ CFU, the protective efficacy of vaccine S1116 (O8) or its combination with other vaccine candidates was therefore not evaluated. Most interestingly, mice vaccinated with mixed equal volumes of S1075 (O9) and S1157 (O7) were able to withstand challenges of *S*. Typhimurium, *S*. Enteritidis, and *S*. Choleraesuis, indicating that a S1075 (O9) and S1157 (O7) co-vaccination strategy may effectively prevent *Salmonella* serotype O4, O9, and O7 infections.

## Discussion

Glycoconjugate vaccines are effective against *Salmonella* spp. infections (10). For instance, Vi-conjugated vaccines can successfully prevent S. Typhi infections (50, 51). O-antigen polysaccharides are usually conjugated to suitable carrier proteins, such as TT, DT, the non-toxic recombinant form of DT (CRM197) (10, 11), and *Salmonella* proteins, to generate glycoconjugate vaccines eliciting T-cell-dependent immune responses with limited memory immunity (26, 27). Usually, this conjugated process is mediated by chemical conjugation, which is an expensive, multiple step process and influenced by various factors such as polysaccharide length and structure. Recently, the discovery of the *Campylobacter jejuni* N-linked and *Neisseria* species O-linked glycosylation pathways paved the way for *in vivo* glycoengineering, the expression of glycosylation machineries in combination with glycan acceptor proteins in heterologous hosts like *E. coli* (52–54). In these two systems, two glycosyltransferases, PglB from *C*. *jejuni* and PglL from *Neisseria*, are critical for functionally transferring polysaccharides to asparagine residues within the glycosylation consensus sequon D/E-X1-N-X2-S/T (X1, X2 ≠ Pro) of acceptor proteins for N-linked and to serine residues within the sequon W-P-Xn-S-Xm-P (Xn is optimal as AAA) for O-linked glycosylation (55–58). The resulting conjugate elicited a robust humoral immunogenic response in animals. Although this technology is used in industrial applications for the generation of glycoconjugated vaccine candidates, there are still some challenges to be overcome, such as limited number of glycosylation consensus sequences on the carrier protein, and poor cytotoxic T lymphocytes development as the glycoconjugates are generally inefficient at entering the class I MHC pathway of antigen presentation (59). Compared to glycoconjugate subunit vaccines, attenuated *Salmonella* possess many advantages in delivering O-antigen polysaccharides including the strong adjuvant effects of *Salmonella* LPS and porins, and the induction of mucosal, humoral, and cellular immune responses with induction of long-term protective immunity (60).

Considering that the invasive NTS are restricted to a limited number of O-antigen serogroups, including B1, D1, C1, and C2 (8, 11), we devised a novel strategy to prevent invasive NTS infections by replacing the original B1 group O-antigen polysaccharide in attenuated *S*. Typhimurium with heterologous O-antigen polysaccharide from *Salmonella* D1, C1, and C2, i.e., immunodominant O-serotype conversion. Our results demonstrated that heterologous O-antigen-specific immune responses could be effectively induced by attenuated *S*. Typhimurium expressing heterologous O-antigen and provide protection against heterologous wild-type virulent *Salmonella* challenge while maintaining homologous protection (Figures 3 and 6).

Our research was initiated by comparing the nucleotide sequences and chemical structures of the O-antigen from serovars of Groups B1, D1, C1, and C2 (Figures S1 and S2 in Supplementary Material). There was high homology in the nucleotide sequences and minor differences in chemical structures between Groups B1 and D1 (61), with an immunodominant abequose (B1) versus tyvelose (D1). Therefore, we converted O4 [group B1, α-Abe(1→3)Man] into O9 [group D1, α-Tyv(1→3)Man] by replacing the allelic *abe* gene with *prt-tyv*_D1_. The LPS profiles of O9 serotype-converted mutants were similar to wild-type *S*. Enteritidis (Figure 2A), indicating that *wzx_B1_*-flippase in *S*. Typhimurium was tolerant to tyvelose side-branch O-units, consistent with a previous report (62). However, the case was more complicated for group C1, as the O-antigen in group C1 serovar starts with GlcNAc initiated by WecA (63, 64). Considering that the nucleotide sequences and chemical structures of group B1 and C1 were completely different, we replaced the entire O-antigen gene cluster of Group B1 with C1 to convert O4 into the O7 serotype. For group C2 serovars, the most important difference lies in additional mannose in the main chain and the α-Abe(1→3)Rha linkages, which consequently contributed to the dominant O8 serotype. Therefore, the genes *wzx*_B1_-*wbaN* were replaced with *wzx*_C2_-*wbaZ* to convert the O4 serotype to O8. LPS silver staining and western blotting confirmed all of these O-serotype conversions (Figure 2). The LPS profile of S1124 (O7) differed from both *S*. Typhimurium and *S*. Choleraesuis, but western blotting using specific anti-O7 serum confirmed that S1124 was able to generate detectable O7 O-antigen polysaccharide, which might indicate subtle decorations on the O-polysaccharide main chain occurred in *S*. Typhimurium (Figure 2B).

*In vitro* analyses indicated that our strategy had resulted in biologically significant changes to the O-antigen. The attachment of bacteriophage P22 to *Salmonella* is mediated by the binding of its tailspike protein to the O-antigenic repeating units of groups B1 and D1 (65). Thus, we obtained transductants using the strain with an engineered group D1 O-antigen, S1031 (O9), while the two strains engineered to produce group C O-antigens were not infected (Table 2). O-antigen is an important factor for swimming or swarming motility on agar surfaces, by improving surface “wettability” (66). Thus, changes in O-antigen composition may have a negative impact on motility. However, our results indicate that the changes we made to O-antigen had no significant impact on motility (Table 2). There were no major changes to the cellular membrane structure, as there was no significant increase in susceptibility to the membrane-damaging agents DOC and polymyxin B (Table 2). There was a slight decrease in the growth rate of strains S1031 (O9), S1124 (O7), and S1131 (O8) compared to their parent strain (Figure S3 in Supplementary Material), and the LD_50_s of S1031 (O9), S1124 (O7), and S1131 (O8) were two orders of magnitude larger than S100 (O4), showing approximately 100-fold attenuation (Table 2). These negative impacts on growth and virulence are likely due to the stress imposed by synthesizing a heterologous O-antigen (67).

Enzyme-linked immunosorbent assay data showed that all the modified vaccines induced a strong heterologous O-antigen-specific serum IgG responses (Figure 3), with IgG2a dominating the anti-LPS response (Figure S6 in Supplementary Material), indicating a Th1-type immune response, consistent with our previous observations (43, 44, 49). Serum antibodies from mice immunized with S738 (O4) and S1075 (O9) were cross-reactive to LPS from *S*. Typhimurium and *S*. Enteritidis, which we ascribed to the shared glycan epitopes O1 or O12. Those from S1157 (O7)- and S1116 (O8)-vaccinated mice were specific to LPS from *S*. Choleraesuis and *S*. Newport, respectively (Figure S7 in Supplementary Material). Moreover, *in vitro* C3 complement deposition and opsonophagocytic assays demonstrated that the vaccine-induced antibodies were able to trigger the classical pathway of complement activation and promote the uptake of wild-type *Salmonella* by macrophages after serum antibody opsonization (Figures 4 and 5). These results underscored that live attenuated *S*. Typhimurium vaccines were able to synthesize heterologous O-antigens on the surface and elicit functional antibody responses in mice targeting the surface polysaccharide antigens of the related wild-type *Salmonella* serovars.

Our goal was to design and construct a *S*. Typhimurium vaccine to provide protection against multiple serovars of NTS infections in addition to *S*. Typhimurium. We observed that all mice survived challenge with 100 times dose of the LD_50_ of *S*. Typhimurium, indicating that the protective efficacy against the homologous parent strain was not compromised, consistent with a study conducted by Hormaeche et al. (33). This may be due to the robust immune responses elicited by all strains against *S*. Typhimurium outer membrane proteins (Figure 3E). This seems likely, as antibody responses to outer membrane proteins have been shown to play an important role in protective immunity against *S*. Typhimurium. For example, immunization of mice with rough *S*. Typhimurium mutants or mutants engineered to shut off O-antigen synthesis *in vivo* elicits protective immunity against lethal challenge with *S*. Typhimurium (22, 23, 43, 44, 49). Immunization with purified outer membrane proteins from rough *S*. Typhimurium elicits long-lasting protective immunity against *S*. Typhimurium challenge (47, 68–70). Some of these outer membrane proteins induce T-cell-mediated immune responses essential for clearance of the bacterial infection (68, 69). In our study, only mice vaccinated with S1075 (O9) or S1157 (O7) survived a challenge with 100 times the LD_50_ of *S*. Enteritidis or *S*. Choleraesuis, respectively, indicating that protective efficacy against heterologous *Salmonella* challenge was highly O-serotype related in this case.

Competition between multiple *Salmonella* serovars in the same host eventually results in the serovar with highest transmission success excluding the other serovars expressing the same O-serotype from the host population (71, 72). This mechanism should not impact our ability to vaccinate a single host with a mixture of our *Salmonella* vaccines, as strains S1075 (O9) and S1157 (O7) each expressed distinct O-antigen polysaccharides. When co-administered, they induced a high level of anti-IgG antibodies against their respective O-antigens (Figure 3). Broad protective coverage of serotypes O4, O9, and O7 was elicited by co-vaccination with S1075 (O9) and S1157 (O7) (Figure 6).

Our wild-type *S*. Newport strain was not virulent in mice (Table 2), so we were unable to evaluate the protective efficacy of S1116 (O8) against a *S*. Newport challenge. We note that the lack of virulence in mice is likely to be due to the absence of a virulence plasmid in *S*. Newport strains (73).

Although we only tested the protective efficacies of one serovars in each serogroup B1, D1, and C1, i.e., *S*. Typhimurium S100, *S*. Enteritidis S246, and *S*. Choleraesuis S340, it is reasonable to predict that vaccination with a serovar producing an immunodominant O-serotype could elicit cross-immunity against members of the same serogroup (74). In summary, we demonstrated that live attenuated *S*. Typhimurium vaccines based on O-serotype conversion were immunogenic and suitable for a co-vaccination strategy to provide protection against serovars *S*. Typhimurium (O4), *S*. Enteritidis (O9), *S*. Choleraesuis (O7), and *S*. Newport (O8), which account for the majority of NTS infections.

## Ethics Statement

All animal studies were conducted in compliance with the Animal Welfare Act and regulations stated in the Guide for the Care and Use of Laboratory Animals, which was approved by Sichuan Agricultural University Institutional Animal Care and Use Committee (Ya’an, China; Approval No. 2011028).

## Author Contributions

PL and QK conceived and designed the experiments. PL, HL, KL, JY, YL, and YH performed the experiments. PL, QL, and YH analyzed the data. PL, QL, and QK wrote the article.

## Conflict of Interest Statement

The authors declare that the research was conducted in the absence of any commercial or financial relationships that could be construed as a potential conflict of interest.

## Supplementary Material

Figure S1 The O-antigen gene clusters of B1, D1, C1, and C2 serogroups. The O-antigen gene clusters of *Salmonella* Typhimurium (B1, O4), *S*. Enteritidis (D1, O9), *S*. Choleraesuis (C1, O7), and *S*. Newport (C2, O8) are shown and the immunodominant O-serotype factor was labeled in front of the gene cluster. Genes were color coded by biosynthesis pathways. Arrows represented the location and orientation of the genes. Diagrams are drawn to scale.

Figure S2 The chemical structures of B1, D1, C1, and C2 serogroup O-units. The chemical structures illustrating the sugar components and glycosidic linkages within the O-units of *Salmonella* Typhimurium **(A)**, *S*. Choleraesuis **(B)**, *S*. Enteritidis **(C)**, and *S*. Newport **(D)**. The immunodominant O-serotype factor was labeled under the structures. Sugar abbreviations: Abe, abequose; Tyv, tyvelose; l-Rha, l-rhamnose; d-Man, d-mannose; d-Gal, d-galactose; d-GlcNAc, d-acetylgalactosamine.

Figure S3 Growth curves of O-serotype converted mutants. *In vitro* growth rates of *Salmonella* Typhimurium wild-type S100, and its derivatives were determined by measurement of the optical density at OD_600_ at multiple time points.

Figure S4 Attachment and invasion assays in Hep-2 cells. The percentage of attachment and invasion rate of O-serotype converted mutants was evaluated as described in materials and methods. All mutants were derived from the S738 (O4) parental strain, as indicated. Error bars represented SEs of the means.

Figure S5 Colonization of murine Peyer’s patches, liver, and spleen by live attenuated *Salmonella* Typhimurium vaccines. All mutants were derived from the S738 (O4) parental strain, as indicated. Colonization of Peyer’s patches **(A)**, liver **(B)**, and spleen **(C)** after 4 and 8 days post-inoculation were shown. The horizontal lines represent the means, and the error bars represent SEs of the means.

Figure S6 Sera IgG1 and IgG2a responses. The responses of IgG isotype subclasses IgG2a and IgG1 were measured in vaccinated mice sera. The LPS of *Salmonella* Typhimurium **(A)**, *S*. Enteritidis **(B)**, *S*. Choleraesuis **(C)**, and *S*. Newport **(D)** were coated in ELISA plates. The data showed the concentrations of IgG1 and IgG2a subclass in mice sera vaccinated by S738 (O4), S1075 (O9), S1157 (O7), and S1116 (O8). All vaccine candidates could induce a significantly higher amount of heterologous LPS-specific IgG2a levels than IgG1 (**, *P* < 0.01). The antibody concentrations were calculated through the standard curve. All concentrations of the measured samples were within the range of the standard curve. Error bars represented the SEs of the means.

Figure S7 Western-blot of *Salmonella* LPS treated by vaccinated mice sera. The primary antibodies used in western-blot were polyclonal antibodies from mice sera vaccinated by S738 (O4) **(A)**, S1075 (O9) **(B)**, S1157 (O7) **(C)**, S1116 (O8) **(D)**, S1075 (O9) plus S1157 (O7) **(E)** and BSG control **(F)**.
