# Supplementary figures and images for: O-Serotype Conversion in Salmonella Typhimurium Induces Protective Immune Responses against Invasive Non-Typhoidal Salmonella Infections
Source: Front Immunol. 2017 Dec 4;8:1647. doi: 10.3389/fimmu.2017.01647 (PMC5722840; doi:10.3389/fimmu.2017.01647)

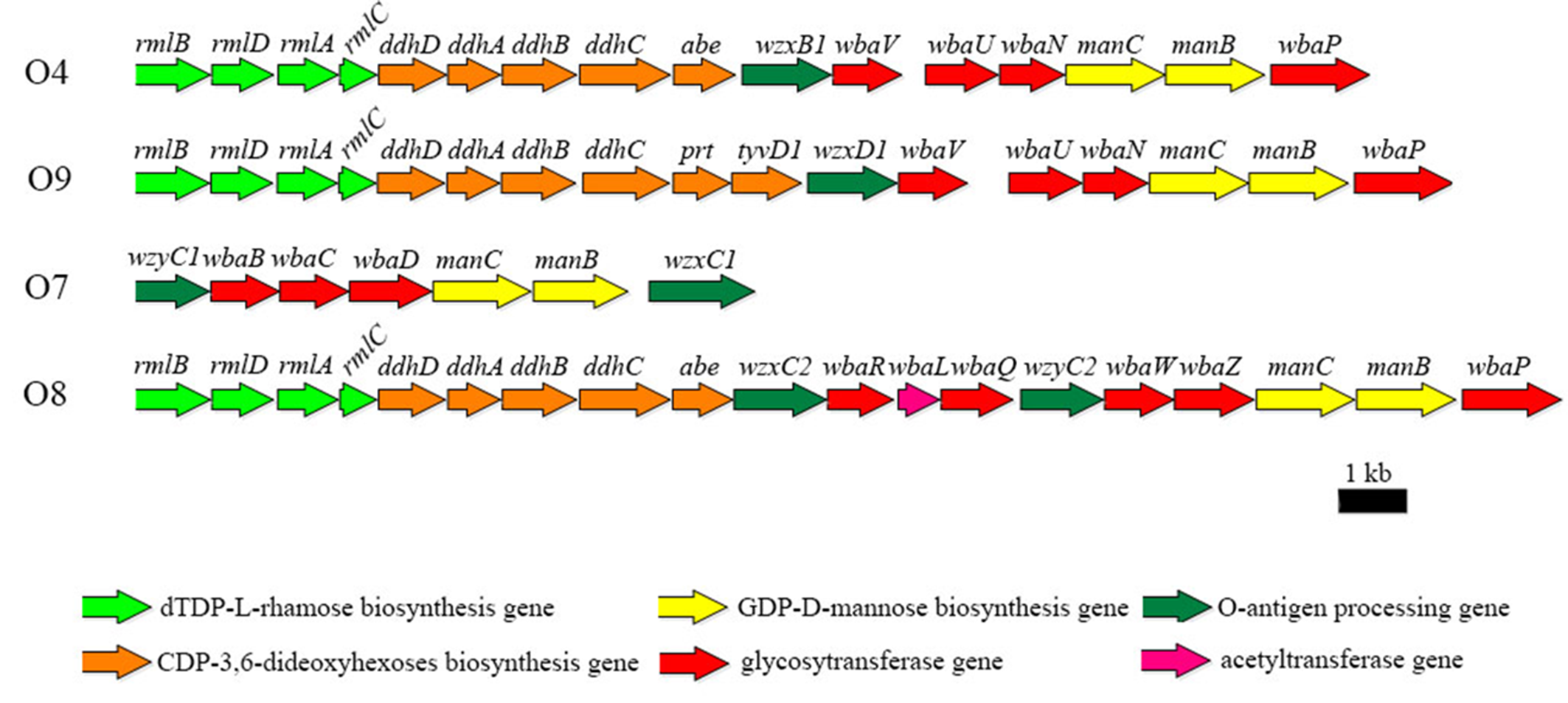

Supplement: Figure S1 — The O-antigen gene clusters of B1, D1, C1, and C2 serogroups. The O-antigen gene clusters of Salmonella Typhimurium (B1, O4), S. Enteritidis (D1, O9), S. Choleraesuis (C1, O7), and S. Newport (C2, O8) are shown and the immunodominant O-serotype factor was labeled in front of the gene cluster. Genes were color coded by biosynthesis pathways. Arrows represented the location and orientation of the genes. Diagrams are drawn to scale. [file Image_1.tif]

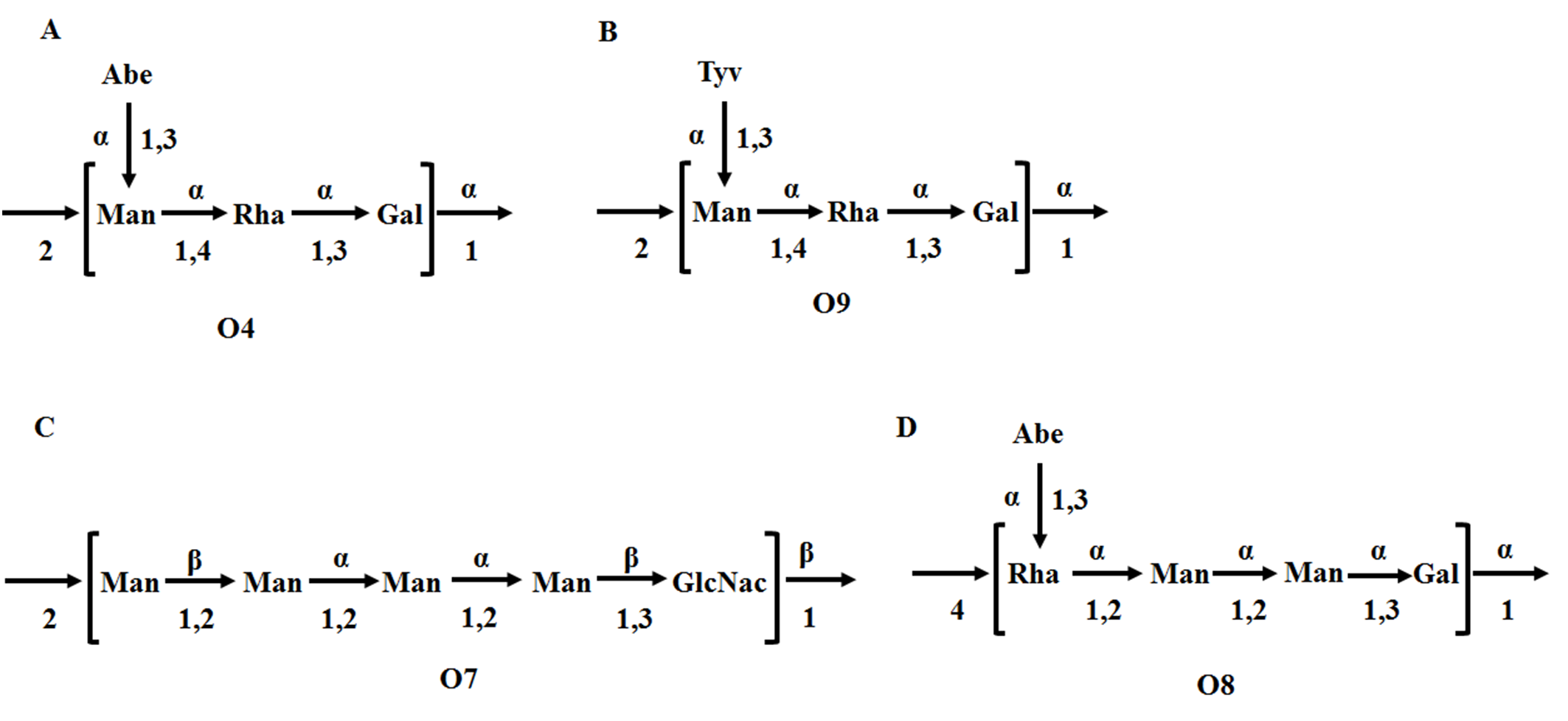

Supplement: Figure S2 — The chemical structures of B1, D1, C1, and C2 serogroup O-units. The chemical structures illustrating the sugar components and glycosidic linkages within the O-units of Salmonella Typhimurium (A), S. Choleraesuis (B), S. Enteritidis (C), and S. Newport (D). The immunodominant O-serotype factor was labeled under the structures. Sugar abbreviations: Abe, abequose; Tyv, tyvelose; l-Rha, l-rhamnose; d-Man, d-mannose; d-Gal, d-galactose; d-GlcNAc, d-acetylgalactosamine. [file Image_2.tif]

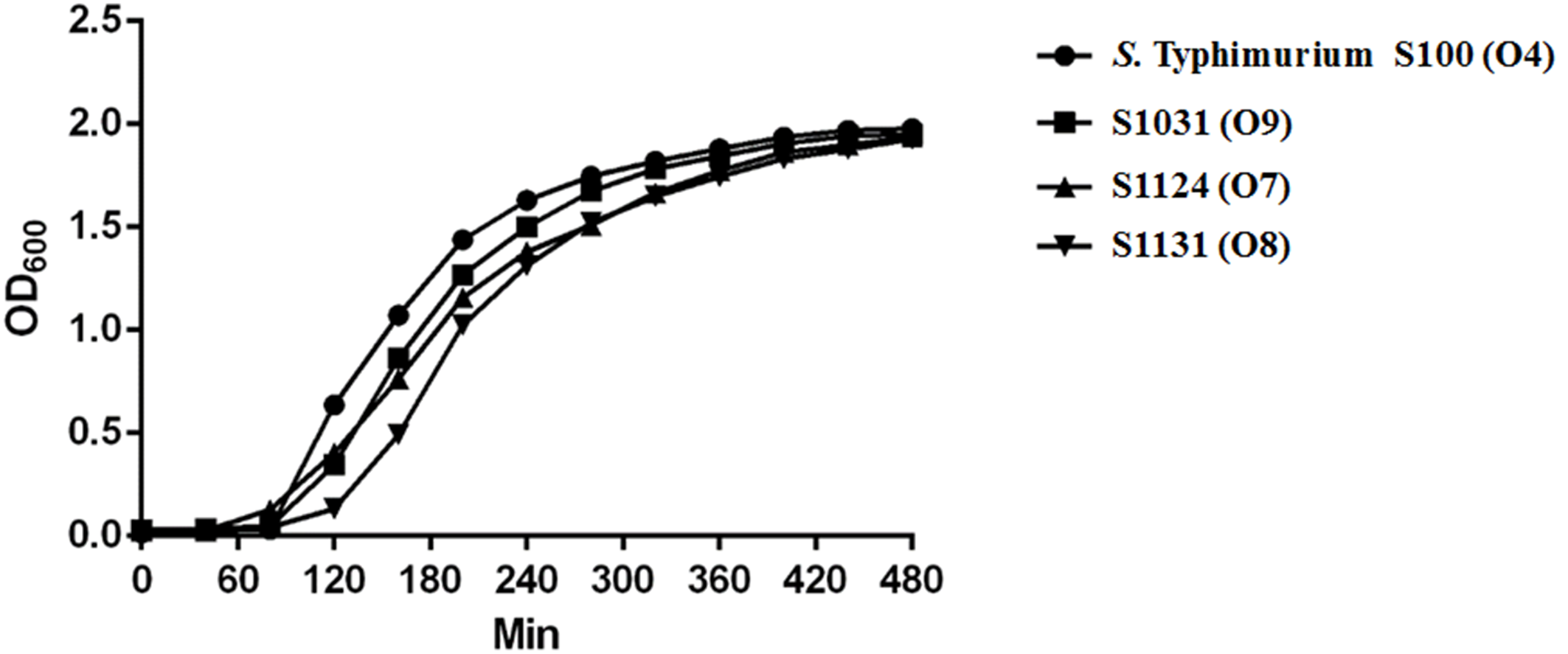

Supplement: Figure S3 — Growth curves of O-serotype converted mutants. In vitro growth rates of Salmonella Typhimurium wild-type S100, and its derivatives were determined by measurement of the optical density at OD600 at multiple time points. [file Image_3.tif]

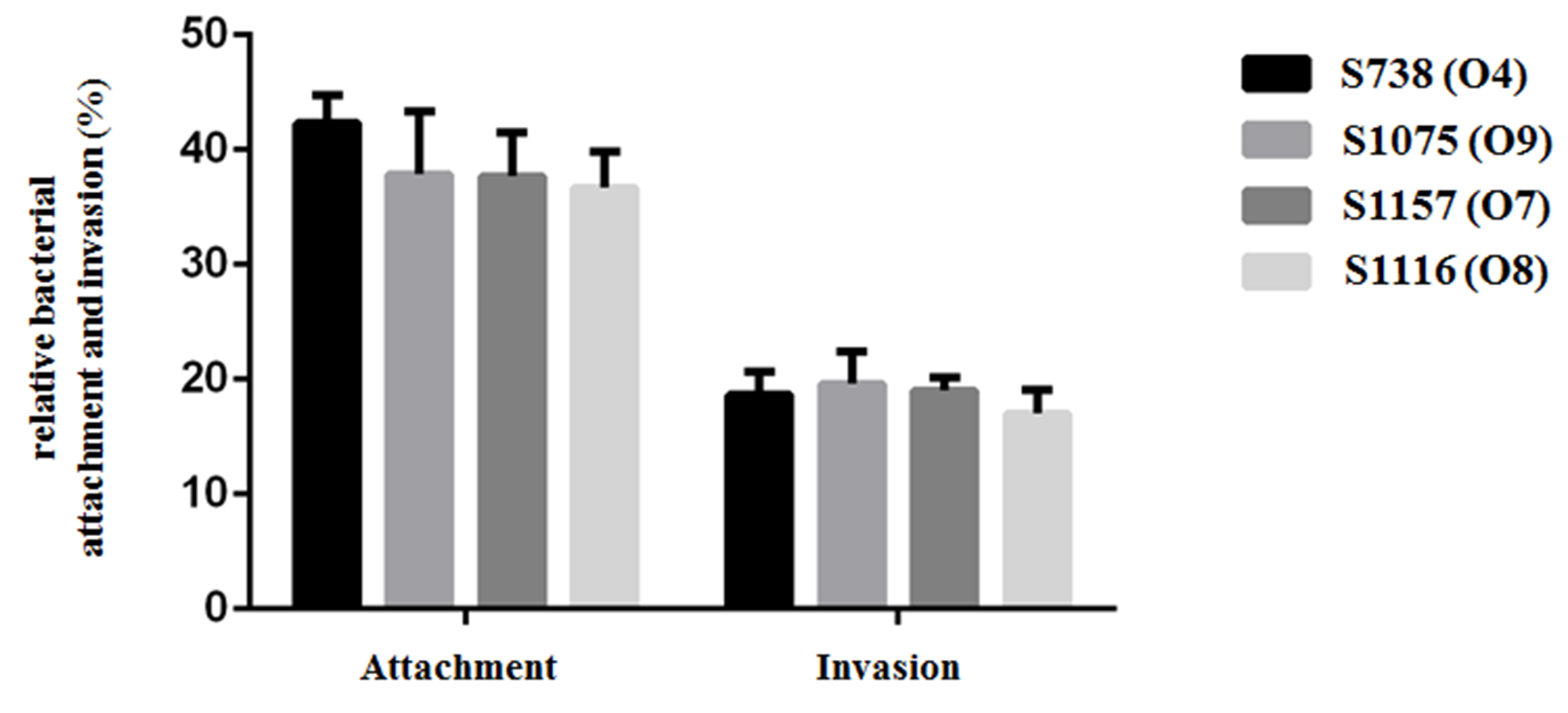

Supplement: Figure S4 — Attachment and invasion assays in Hep-2 cells. The percentage of attachment and invasion rate of O-serotype converted mutants was evaluated as described in materials and methods. All mutants were derived from the S738 (O4) parental strain, as indicated. Error bars represented SEs of the means. [file Image_4.tif]

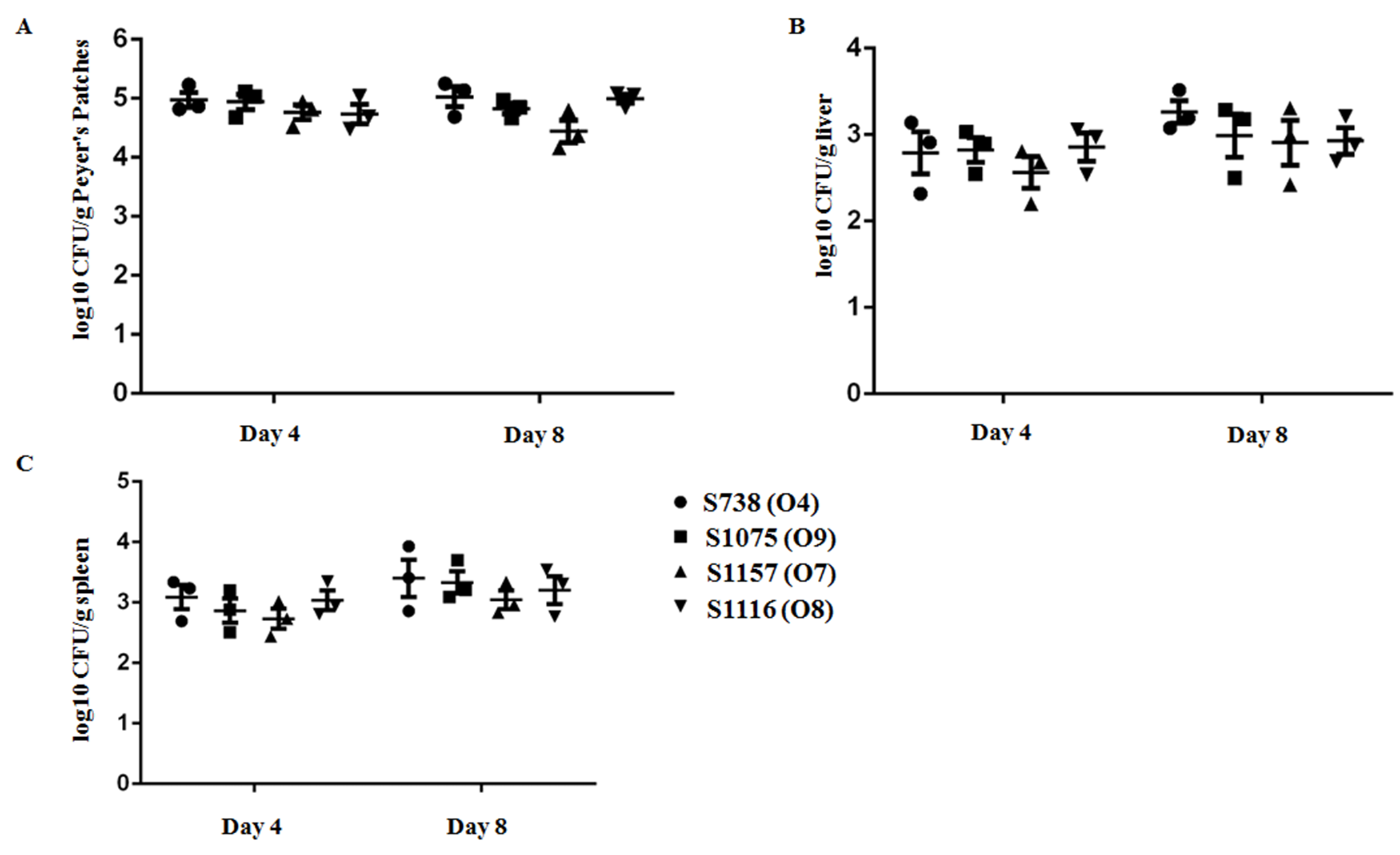

Supplement: Figure S5 — Colonization of murine Peyer’s patches, liver, and spleen by live attenuated Salmonella Typhimurium vaccines. All mutants were derived from the S738 (O4) parental strain, as indicated. Colonization of Peyer’s patches (A), liver (B), and spleen (C) after 4 and 8 days post-inoculation were shown. The horizontal lines represent the means, and the error bars represent SEs of the means. [file Image_5.tif]

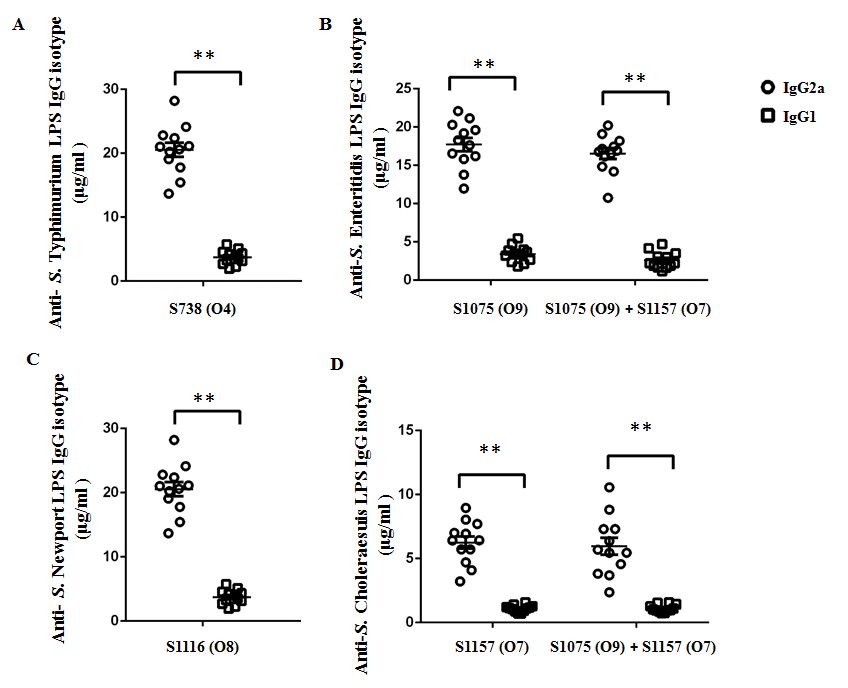

Supplement: Figure S6 — Sera IgG1 and IgG2a responses. The responses of IgG isotype subclasses IgG2a and IgG1 were measured in vaccinated mice sera. The LPS of Salmonella Typhimurium (A), S. Enteritidis (B), S. Choleraesuis (C), and S. Newport (D) were coated in ELISA plates. The data showed the concentrations of IgG1 and IgG2a subclass in mice sera vaccinated by S738 (O4), S1075 (O9), S1157 (O7), and S1116 (O8). All vaccine candidates could induce a significantly higher amount of heterologous LPS-specific IgG2a levels than IgG1 (**, P < 0.01). The antibody concentrations were calculated through the standard curve. All concentrations of the measured samples were within the range of the standard curve. Error bars represented the SEs of the means. [file Image_6.tif]

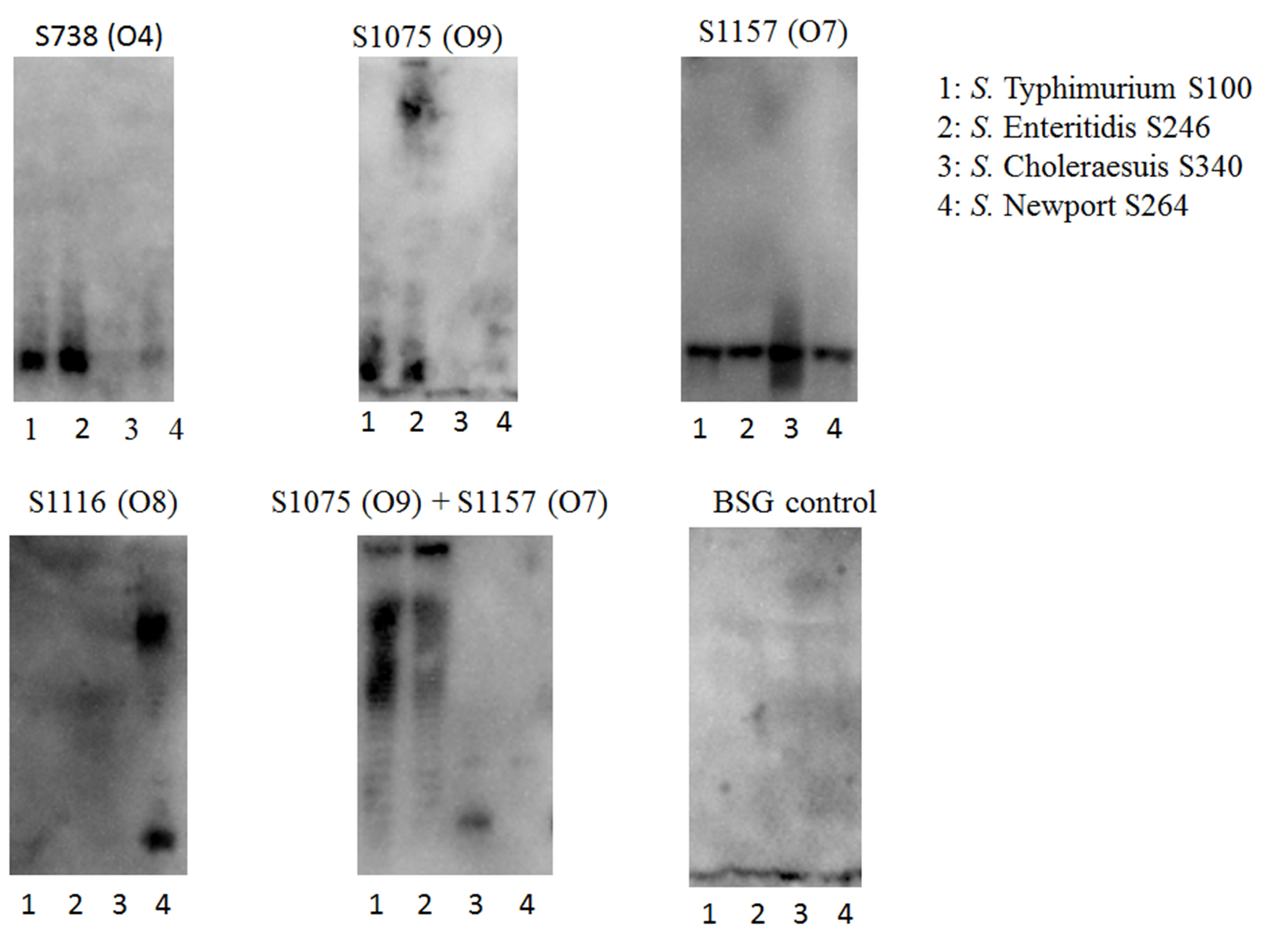

Supplement: Figure S7 — Western-blot of Salmonella LPS treated by vaccinated mice sera. The primary antibodies used in western-blot were polyclonal antibodies from mice sera vaccinated by S738 (O4) (A), S1075 (O9) (B), S1157 (O7) (C), S1116 (O8) (D), S1075 (O9) plus S1157 (O7) (E) and BSG control (F). [file Image_7.tif]
