# Supplementary material for: O-Serotype Conversion in Salmonella Typhimurium Induces Protective Immune Responses against Invasive Non-Typhoidal Salmonella Infections
Source: Front Immunol. 2017 Dec 4;8:1647. doi: 10.3389/fimmu.2017.01647 (PMC5722840; doi:10.3389/fimmu.2017.01647)
Supplement: Supplementary file 8 [file Table_1.docx]

**Table S1. Primers used in this work**

| Primer name | Sequence 5'-3' |
| --- | --- |
| D-abe-1F | gtggtctggctcagttgg |
| D-abe-1R | catgaaaatccagatagaataaattgaattagaaattcaaaccaaag |
| D-abe-2F | atttattctatctggattttcatgatcttttaataaat |
| D-abe-2R | gtttttggctctgcattctgatat |
| (G)Vec-D-abe-F | actagttctatatgaatcgttaacaaattagtcgcgttatg |
| (G)Vec-D-abe-R | ttcatcgcaatcaccagatagaataaattgaattagaaattc |
| (G)In-(prt-tyv)-F | tttattctatctggtgattgcgatgaaaattctaataatgggagcg |
| (G)In-(prt-tyv)-R | taatttgttaacgattcatatagaactagtccaatcatac |
| D-(rmlB-wbaP)-1F | gttggctgaaacgagtgttg |
| D-(rmlB-wbaP)-1R | ggtaagcgcggccgcttttctattccataaggcgt |
| D-(rmlB-wbaP)-2F | tagaaaagcggccgcgcttaccgagaagtactgaat |
| D-(rmlB-wbaP)-2R | ggcaccagctttttgccggg |
| (G)Vec-D-(rmlB-wbaP)-F | gtggtattttaagcttaccgagaagtactgaata |
| (G)Vec-D-(rmlB-wbaP)-R | ataatatatcatttttctattccataaggcgtat |
| (G)In-(wzyC1-wzxC1)-F | gaatagaaaaatgatatattatatttttattg |
| (G)In-(wzyC1-wzxC1)-R | ctcggtaagcttaaaataccacgcgtaaaattc |
| D-(wzxB1-wbaN)-1F | cactttgatgaaattgggc |
| D-(wzxB1-wbaN)-1R | tatctcttttttagtttttacaacataacgcgac |
| D-(wzxB1-wbaN)-2F | gtaaaaactaaaaaagagataaaataaatgtc |
| D-(wzxB1-wbaN)-2R | ggaacaataccaaaagttacaag |
| (G)Vec-D-(wzxB1-wbaN)-F | tattaaacgttaagagataaaataaatgtcttttcttcccg |
| (G)Vec-D-(wzxB1-wbaN)-R | aatacgattcattatgcaattaaacgacttagtttttac |
| (G)In-(wzxC2-wbaZ)-F | tttaattgcataatgaatcgtattattagaatgttaggtgtag |
| (G)In-(wzxC2-wbaZ)-R | ttattttatctcttaacgtttaataatctgttctgttttgaaaag |
